# Key questions in marine mammal bioenergetics

**DOI:** 10.1093/conphys/coac055

**Published:** 2022-08-06

**Authors:** Elizabeth A McHuron, Stephanie Adamczak, John P Y Arnould, Erin Ashe, Cormac Booth, W Don Bowen, Fredrik Christiansen, Magda Chudzinska, Daniel P Costa, Andreas Fahlman, Nicholas A Farmer, Sarah M E Fortune, Cara A Gallagher, Kelly A Keen, Peter T Madsen, Clive R McMahon, Jacob Nabe-Nielsen, Dawn P Noren, Shawn R Noren, Enrico Pirotta, David A S Rosen, Cassie N Speakman, Stella Villegas-Amtmann, Rob Williams

**Affiliations:** Cooperative Institute for Climate, Ocean, and Ecosystem Studies, University of Washington, Seattle, WA, 98195, USA; Ecology and Evolutionary Biology Department, University of California Santa Cruz, Santa Cruz, CA, 95064, USA; School of Life and Environmental Sciences, Deakin University, Burwood, VIC 3125, Australia; Oceans Initiative, Seattle, WA, 98102, USA; SMRU Consulting, Scottish Oceans Institute, University of St. Andrews, St. Andrews KY16 8LB, UK; Biology Department, Dalhousie University, Halifax, NS B3H 4R2, Canada; Population Ecology Division, Bedford Institute of Oceanography, Dartmouth, NS B2Y 4A2, Canada; Aarhus Institute of Advanced Studies, 8000 Aarhus C, Denmark; Zoophysiology, Department of Biology, Aarhus University, 8000 Aarhus C, Denmark; Center for Sustainable Aquatic Ecosystems, Harry Butler Institute, Murdoch, Murdoch University, WA 6150, Australia; SMRU Consulting, Scottish Oceans Institute, University of St. Andrews, St. Andrews KY16 8LB, UK; Sea Mammal Research Unit, Scottish Oceans Institute, University of St. Andrews, St. Andrews KY16 9XL, UK; Ecology and Evolutionary Biology Department, University of California Santa Cruz, Santa Cruz, CA, 95064, USA; Fundación Oceanogràfic de la Comunitat Valenciana, 46005 Valencia, Spain; Kolmården Wildlife Park, 618 92 Kolmården, Sweden; NOAA/National Marine Fisheries Service, Southeast Regional Office, St. Petersburg, FL, 33701, USA; Department of Oceanography, Dalhousie University, Halifax, NS B3H 4R2, Canada; Plant Ecology and Nature Conservation, University of Potsdam, 14476 Potsdam, Germany; Ecology and Evolutionary Biology Department, University of California Santa Cruz, Santa Cruz, CA, 95064, USA; Zoophysiology, Department of Biology, Aarhus University, 8000 Aarhus C, Denmark; IMOS Animal Tagging, Sydney Institute of Marine Science, Mosman, NSW 2088, Australia; Department of Ecoscience, Aarhus University, 4000 Roskilde, Denmark; Conservation Biology Division, Northwest Fisheries Science Center, National Marine Fisheries Service, National Oceanic and Atmospheric Administration, Seattle, WA, 98112, USA; Institute of Marine Science, University of California Santa Cruz, Santa Cruz, CA, 95060, USA; Centre for Research into Ecological and Environmental Modelling, University of St. Andrews, St. Andrews KY16 9LZ, UK; Institute for Oceans and Fisheries, University of British Columbia, Vancouver, BC V6T 1ZA, Canada; School of Life and Environmental Sciences, Deakin University, Burwood, VIC 3125, Australia; Ecology and Evolutionary Biology Department, University of California Santa Cruz, Santa Cruz, CA, 95064, USA; Oceans Initiative, Seattle, WA, 98102, USA

## Abstract

Bioenergetic approaches are increasingly used to understand how marine mammal populations could be affected by a changing and disturbed aquatic environment. There remain considerable gaps in our knowledge of marine mammal bioenergetics, which hinder the application of bioenergetic studies to inform policy decisions. We conducted a priority-setting exercise to identify high-priority unanswered questions in marine mammal bioenergetics, with an emphasis on questions relevant to conservation and management. Electronic communication and a virtual workshop were used to solicit and collate potential research questions from the marine mammal bioenergetic community. From a final list of 39 questions, 11 were identified as ‘key’ questions because they received votes from at least 50% of survey participants. Key questions included those related to energy intake (prey landscapes, exposure to human activities) and expenditure (field metabolic rate, exposure to human activities, lactation, time-activity budgets), energy allocation priorities, metrics of body condition and relationships with survival and reproductive success and extrapolation of data from one species to another. Existing tools to address key questions include labelled water, animal-borne sensors, mark-resight data from long-term research programs, environmental DNA and unmanned vehicles. Further validation of existing approaches and development of new methodologies are needed to comprehensively address some key questions, particularly for cetaceans. The identification of these key questions can provide a guiding framework to set research priorities, which ultimately may yield more accurate information to inform policies and better conserve marine mammal populations.

## Teaser

Marine mammals are facing many threats that impact their ability to obtain energy to survive and reproduce. Much remains unknown about marine mammal bioenergetics, which hinders real-world applications to conservation and management. We surveyed the bioenergetic community to identify ‘key’ unanswered questions to help guide future marine mammal research efforts.

## Introduction

Bioenergetics is the study of the acquisition and allocation of energy by individuals to support maintenance, activity, growth and reproduction (Lavigne *et al.,* 1982). It is an integral component of conservation physiology, which aims to understand and predict how organisms, populations and ultimately ecosystems respond to environmental variation and stressors (Cooke *et al.,* 2013). Many of the conservation challenges facing marine mammals today revolve around bioenergetics, such as environmental variability, climate change, fisheries interactions, predation risk, offshore development, and noise pollution (Wirsing *et al.,* 2008; Davidson *et al.,* 2012; Kovacs *et al.,* 2012; Avila *et al.,* 2018). For example, climate change may alter an individual’s energy intake through changes in prey distribution, abundance, and energy density (von Biela *et al.,* 2019; Gallagher *et al.,* 2022). It can also affect energy expenditure through changes in habitat availability (Pagano and Williams, 2021) or thermal landscapes (Cunningham *et al.,* 2021). Evidence is mounting that the inability to obtain sufficient energy is affecting individual growth, reproduction, and survival of marine mammals (Stirling and Derocher, 2012; Ferguson *et al.,* 2017; Christiansen *et al.,* 2020, 2021; Stewart *et al.,* 2021a). If a sufficiently large number of individuals are affected, this can ultimately affect population growth rates (New *et al.,* 2014; Baylis *et al.,* 2015), as illustrated by the Population Consequences of Disturbance (PCoD) framework (Pirotta *et al.,* 2018a).

The first comprehensive review of marine mammal bioenergetics occurred at the Mammals in the Seas conference organized by the United Nations Food and Agriculture Organization in 1976 (Lavigne *et al.,* 1982). The impetus for this effort was an interest in quantifying the impacts of marine mammals on commercially valuable fish populations. This review stimulated studies that examined the general patterns of energetics at the population level (Lockyer, 1981; Lavigne, 1982), feeding rates of marine mammals (Innes *et al.,* 1987), and a review of metabolic rates (Lavigne *et al.,* 1986). Many of these papers challenged early hypotheses that marine mammals have higher metabolic rates than would be predicted for similarly sized terrestrial mammals (Scholander *et al.,* 1950; Kanwisher and Ridgway, 1983), a topic still debated today (Nagy *et al.,* 1999; Hunter *et al.,* 2000; Williams and Yeates, 2004; Costa and Maresh, 2017; Williams *et al.,* 2020). A symposium in 1985 highlighted advances in our understanding of the energetics of marine mammals and technological developments (Huntley *et al.,* 1987). Our knowledge of energetics has continued to grow, as have the implications and applications to conservation and management issues. Recently, there has been a resurgence in the use of bioenergetic modelling approaches to quantify predator–prey interactions (e.g. Fortune *et al.,* 2013; Chasco *et al.,* 2017; McHuron *et al.,* 2020; Acevedo and Urbán, 2021) and predict the individual- and population-level effects of altered environments on marine mammals (e.g. Christiansen and Lusseau, 2015; Udevitz *et al.,* 2017; Nabe-Nielsen *et al.,* 2018; Farmer *et al.,* 2018b; Pirotta *et al.,* 2019; Gallagher *et al.,* 2020; Silva *et al.,* 2020; Gavrilchuk *et al.,* 2021). Technological and analytical advances have furthered these empirical and modelling methodologies (Pirotta, 2022).

Despite recent advances, the field is still hindered by many of the same uncertainties and data deficiencies that existed nearly four decades ago, especially for cetaceans. As it is not feasible to address these deficiencies for all 127 extant marine mammal species, research priorities must be identified based on the perceived needs of the bioenergetics community. To achieve this, increased communication within the bioenergetic community is needed to align data needs and data generation. Our aim was to identify ‘key’ outstanding questions in the field of marine mammal bioenergetics. The intent is to stimulate and focus research that will most effectively lead to an increased understanding of the ecology and population biology of marine mammals, ultimately facilitating conservation and management efforts. This effort was conducted in tandem with a bioenergetics workshop to discuss and review the current state of knowledge on marine mammal bioenergetics.

## Methods

**Figure 1:**
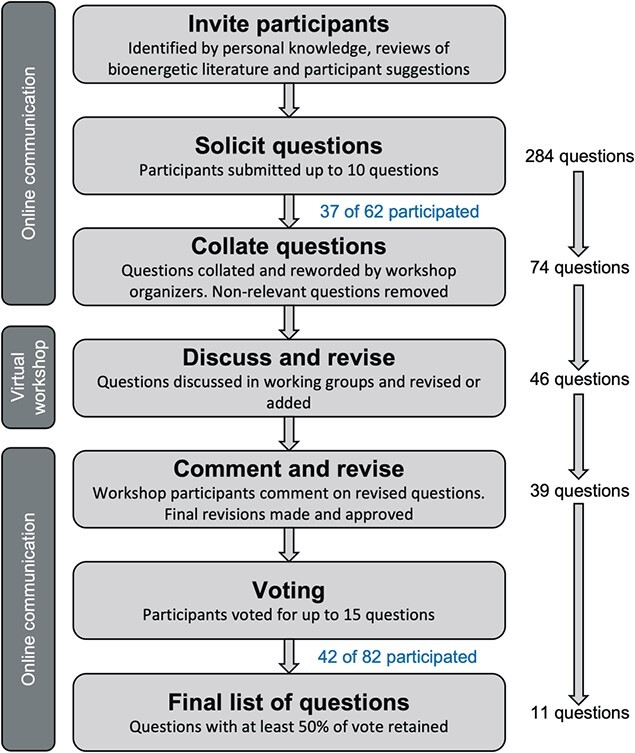
Process for identifying key bioenergetic questions. Figure after Sutherland et al. (2022). The number of participants compared with invitees is shown in blue text for the initial question submission and the voting on final questions. The number of invitees increased from 62 to 82 due to additional participant suggestions by co-authors.

We invited 62 scientists with experience in marine mammal physiology, bioenergetics, trophic ecology and population dynamics, to identify unanswered questions in marine mammal bioenergetics. The participant list was generated by the workshop organizers with additional suggestions from invited participants. Identification of participants by workshop organizers was based on personal knowledge of participants’ research and queries of the relevant bioenergetic and marine mammal literature. Participants were asked to submit no more than 10 questions each with the following guidelines. Questions (i) could not be answered with a simple ‘yes’, ‘no’ or ‘depends’; (ii) could be related to any aspect of bioenergetics but should be applicable to methods used in marine mammal management and conservation; (iii) could be unanswerable or infeasible given current methods; and (iv) could be either species-specific or broadly relevant to a taxonomic group (e.g. pinnipeds, cetaceans). Questions were collated and revised over email discussions and during the bioenergetic workshop (Fig. 1). Questions that were not directly related to bioenergetics were removed from further consideration.

The final list of questions was circulated for voting to the original 62 survey invitees and 20 additional individuals identified by the co-authors of this paper during the process. Participants voted for no more than 15 questions each, with the guidance that a question need not be applicable to all marine mammals (i.e. it might only be important or unanswered for certain species, taxonomic groups or species with similar life history strategies). Voting occurred virtually via a Google Forms survey (25 August–27 September 2021). Survey participants were also asked to provide information on their expertise in marine mammal physiology and whether they had experience developing bioenergetic models (each on a scale of 1–10, from least to most experience). This was to help inform whether any key questions were deemed important by individuals with greater expertise in one discipline over the other (physiologists vs. bioenergetic modellers).

## Results and discussion

A total of 284 initial questions were submitted by 37 of the 62 original invited participants. They ranged from detailed physiological questions to broad overarching questions about the impacts of climate change, anthropogenic disturbance and fisheries on marine mammal populations. Questions were revised by co-authors and other bioenergetic workshop participants (Fig. 1) such that detailed physiological questions fell under the umbrella of more general questions. This ensured that final questions were not too narrow in scope. Broad overarching questions were not explicitly included in the final question list because numerous research priorities were needed to address such broad questions, and there was often overlap in research priorities among these questions (i.e. the same research priorities were needed to address a diversity of broad questions). Instead, the final list of questions reflected these individual research priorities. Collation resulted in 39 final questions (Fig. 1). A complete list of the initial and final questions can be found in Tables S1 and S2.

**Figure 2:**
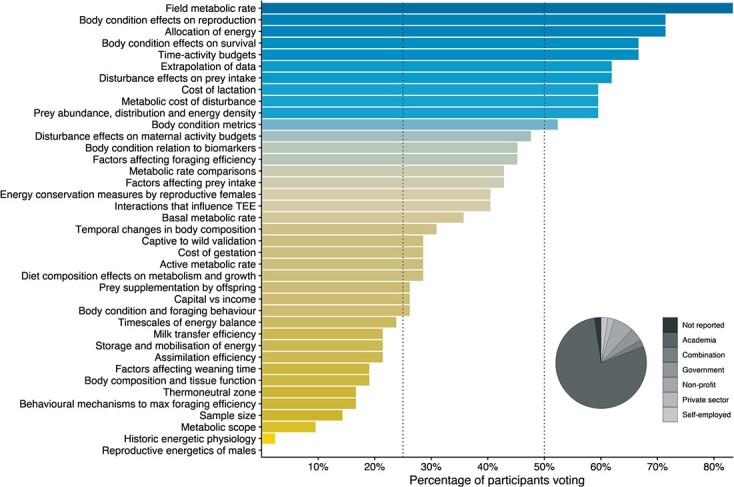
The percentage of survey participants that voted for each of the 39 questions, ordered (and coloured) from least to greatest number of votes. Key questions were defined as those voted by at least 50% of participants. The inset pie graph shows the self-reported employment type of participants that voted in the final survey. The full phrasing of each question can be found in Fig. 3 and Table S2.

Forty-two of the 82 (62 original +20 additional) invited participants voted in the final survey. Most of the voting participants had current jobs in academia (78%), followed by non-profits (7.1%), government agencies (4.8%), a combination of academia and consulting (2.4%), the private sector (2.4%) or were self-employed (2.4%; Fig. 2). One participant declined to include their affiliation. Self-assigned scores for physiological and bioenergetic modelling knowledge (on a scale of 1–10) ranged from 3–10 (average of 7.3) and 2–10 (average of 6.8), respectively. There were 11 questions that at least 50% of the participants voted for, 16 questions that 25–50% of the participants voted for and 12 questions that received <25% of votes (Fig. 2). We selected those questions that received ≥50% of votes as key questions. Average expertise scores for key questions were similar (absolute differences of 0.06–1.0 on a scale of 1–10), suggesting broad agreement within the community about research priorities.

**Figure 3:**
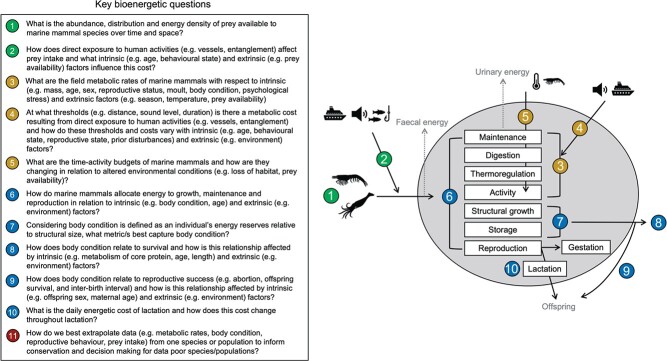
The key outstanding bioenergetic questions identified as part of this exercise (left) and a conceptual diagram of where each question (excluding Q11) fits in the energy flow through an animal (grey circle). Questions are coloured based on the general categories of prey intake (green), energy expenditure on self (gold) and energy allocation and storage for other processes, such as reproduction (blue).

The following sections briefly describe how each question is relevant to bioenergetics and conservation efforts and highlight important data gaps. Additional information on topics covered here, such as bioenergetic models, metabolic rates, body condition and growth and reproductive energetics can be found elsewhere in this special issue (also see reviews by Iverson *et al.,* 2010; Rosen and Worthy, 2018; Watanabe and Goldbogen, 2021). We conclude by discussing some of the existing methodologies available to address key questions and areas where further methodological advancements are needed. The headers below have been paraphrased from the key questions (Fig. 3) and in some cases encompass more than one key question for brevity. As we did not rank key questions in terms of importance, the order of questions below follows the numerical order from Fig. 3.

### What is the abundance, distribution and energy density of prey? (Q1)

Knowledge of the abundance, distribution and energy density of prey in time and space is critical to answering a variety of bioenergetic questions. For example, is there enough prey biomass (of the right type) in the right place, at the right time to support individual and population energy needs? How do factors such as interannual variability, climate change, fisheries and habitat loss affect prey availability? Climate change, which is rapid in some regions, is predicted to have considerable impacts on the distribution, biomass, energy density and body size of prey species (Flores *et al.,* 2012; Yasumiishi *et al.,* 2020; Florko *et al.,* 2021). Such changes have clear bioenergetic implications for marine mammals and the ecosystems they inhabit (Costa, 2008; Laidre *et al.,* 2020; Gallagher *et al.,* 2022). Since the prey landscape is a major driver of the spatiotemporal distribution of marine mammals (Sveegaard *et al.,* 2012; Zerbini *et al.,* 2016; Sigler *et al.,* 2017; Straley *et al.,* 2018; Pendleton *et al.,* 2020), knowledge of prey fields and how they may be changing provides insight into the potential impact of anthropogenic disturbances on energy budgets (Keen *et al.,* 2021). As such, prey fields are critical components of many PCoD models (Nabe-Nielsen *et al.,* 2018; Pirotta *et al.,* 2019; McHuron *et al.,* 2021). At finer temporal and spatial scales (e.g. within a prey patch), measurements of the prey landscape can inform relationships between prey density, foraging effort and energy gain (Bowen *et al.,* 2002; Hazen *et al.,* 2015; Cade *et al.,* 2021). Even in well-studied ecosystems, there are very few (if any) marine mammal species for which the prey landscape (including energy density of prey species) has been sufficiently resolved to predict behaviour and fine-scale spatial distribution through time.

### At what thresholds do disturbance from human activities affect energy intake and expenditure? (Q2, Q4)

Direct exposure of marine mammals to human activities can elicit behavioural responses (e.g. changes in foraging behaviour) or cause direct (e.g. injuries from vessels or nets) or indirect (e.g. pollution, physiological effects) physical impacts that have implications for energy balance. For example, marine mammals may spend less time foraging when disturbed (Senigaglia *et al.,* 2016; Harris *et al.,* 2018), which could reduce energy intake. At the same time, a disturbance may alter energy expenditure if it elicits a strong physiological response (Christiansen *et al.,* 2014a; Williams *et al.,* 2017) or causes a switch to activities that have greater energetic costs, such as the increase in surface activity exhibited by some delphinids when exposed to vessels (Coscarella *et al.,* 2003; Lusseau, 2006; Noren *et al.,* 2009, 2012). Traumatic stressors, such as non-lethal entanglement or vessel strikes, can impact energy budgets by altering movement costs, foraging behaviour or energy investment in tissue healing and regrowth (Wells *et al.,* 2008; van der Hoop *et al.,* 2017; Pettis *et al.,* 2017), or cause permanent energetic changes from severe injuries (e.g. amputation). While not a classical disturbance, exposure to contaminants has been linked with metabolic disruptions in grey seals (*Halichoerus grypus*), leading to reduced weaning mass of pups (Robinson *et al.,* 2018; Bennett *et al.,* 2021). This key question is the logical next step in ongoing research into the behavioural responses of marine mammals to disturbance, as it addresses the thresholds (e.g. duration, severity) that induce behavioural responses that are energetically meaningful to an individual and thus have the potential for population-level consequences. When assessing thresholds, it is important to consider the extent and timescales at which an animal can compensate (Hin *et al.,* 2019; Pirotta *et al.,* 2019; Booth, 2020). Our ability to accurately quantify the energetic implications of disturbance in part relies on addressing other key questions (Q1 on prey landscapes and Q3 on metabolic rates).

### What are the field metabolic rates of marine mammals? (Q3)

Field metabolic rates (FMRs) represent an individual’s daily energy expenditure at a given time, which underpins many of the other key questions we identified in this exercise. The costs contained within FMR comprise the majority of an individual’s energy budget (Winship *et al.,* 2002; Rechsteiner *et al.,* 2013; Bejarano *et al.,* 2017; McHuron *et al.,* 2020). Marine mammal FMRs can vary with behavior, season, age class and among species (Arnould *et al.,* 1996b; Costa and Gales, 2000; Mellish *et al.,* 2000; Bowen *et al.,* 2001; Trillmich and Kooyman, 2001; Costa and Gales, 2003; Fowler *et al.,* 2007; Costa, 2008; Villegas-Amtmann *et al.,* 2017a; Jeanniard du Dot *et al.,* 2018; McHuron *et al.,* 2018; Rojano-Donãte *et al.,* 2018; McHuron *et al.,* 2019). Otariids generally have elevated FMRs compared with those of phocids (Costa and Maresh, 2017), and limited data from bottlenose dolphins (*Tursiops truncatus*) and harbour porpoise (*Phocoena phocoena*) indicate that FMRs of small odontocetes may align more closely with the high-energy lifestyle of otariids (Costa and Maresh, 2017; Rojano-Donãte *et al.,* 2018). Due to the logistical challenges in measuring FMR in free-ranging marine mammals, most existing measurements are from pinnipeds during lactation. Considerable data gaps remain for many marine mammals, particularly deep-diving beaked (*Ziiphidae*) and sperm whales (*Physeter macrocephalus*) and baleen whales. These gaps often result in the use of general allometric equations to estimate metabolic rates in bioenergetic models (e.g. Bejarano *et al.,* 2017; Acevedo and Urbán, 2021). Existing intra- and inter-group-specific differences in marine mammal FMRs, and evidence that there is heterogeneity in allometric scaling factors with body mass and taxonomy (McNab, 2008; Kolokotrones *et al.,* 2010; Hudson *et al.,* 2013), highlight the need for additional FMR data.

### What are the time-activity budgets of individuals and how are they changing in response to altered environmental conditions? (Q5)

The amount of time an animal spends engaged in certain activities (e.g. resting, foraging, travelling, breeding/socialising) influences its energy budget through changes in intake, expenditure or both. Climate change and habitat loss alter environmental conditions, forcing (or facilitating) species to either move into new environments or adapt to their existing ones that may be undergoing rapid changes (Silber *et al.,* 2017; Pinsky *et al.,* 2020). Effects of climate change on time-activity budgets have been documented for some marine mammals (Hamilton *et al.,* 2018; Blanchet *et al.,* 2020). While time-activity budgets have been estimated for numerous species, new studies are needed to capture responses to recent environmental changes at varying temporal scales, as environmental variation may influence activity budgets at some scales and not others (Austin *et al.,* 2006). A better understanding of activity-specific metabolic costs is then needed to assess how such changes affect energy expenditure, although this question did not receive enough votes to be classified as a key question in this exercise (Fig. 2). In addition to assessing effects on energy balance, understanding time-activity budgets can also provide insight into a species’ flexibility to respond to environmental perturbations. For example, near-continuous foraging in northern elephant seals (*Mirounga angustirostris*; Adachi *et al.,* 2021), harbour porpoises (Wisniewska *et al.,* 2016) and sperm whales (Watwood *et al.,* 2006; Farmer *et al.,* 2018a) indicate these species may have little flexibility to adjust to reductions in food availability or interruptions in foraging (but see Hoekendijk *et al.,* 2018; Booth, 2020).

### How do marine mammals allocate energy to maintenance, growth and reproduction? (Q6)

Once ingested, energy is directed to a variety of processes, such as digestion, the maintenance of cellular function, production of waste products, thermoregulation, mechanical work/activity, structural growth, reproduction and storage (Fig. 3). Energy allocation must be prioritised when energy intake is insufficient to fuel all these costs (see review by Glazier, 2009). This prioritisation exists at different hierarchical levels, from partitioning among processes (e.g. maintenance vs. growth) to partitioning among organs or tissues within individual compartments. In general, energy allocation to maintenance is prioritised before growth and reproduction (Costa *et al.,* 1989; Soto *et al.,* 2004; Wheatley *et al.,* 2006; Christiansen *et al.,* 2014b, 2018; Kershaw *et al.,* 2021; Smith, 2021). Compensatory mechanisms, such as metabolic depression, may help cope with energy limitation (Markussen *et al.,* 1992; Rosen and Trites, 2002) or periods of high energy demand (Mellish *et al.,* 2000; Shuert *et al.,* 2020), although little is known about the drawbacks of such mechanisms (Halsey, 2021). Understanding these priorities is important as many bioenergetic models use researcher-defined rules regarding allocation when energy intake is insufficient to meet an individual’s needs, such as when foraging may be disrupted by a disturbance (e.g. Villegas-Amtmann *et al.,* 2017b; Farmer *et al.,* 2018b). Reduced allocation to reproduction could lead to changes in offspring body size that persist across an individual’s lifetime, an issue recently highlighted for North Atlantic right whales (*Eubalaena glacialis*; Stewart *et al.,* 2021b). Understanding when reduced energy allocation to growth may occur, and the magnitude of such reduction, is critical since smaller body size could have wide-ranging impacts on reproductive behaviour and success of many marine mammals.

### What metrics best capture body condition? (Q7)

Body condition, defined as the amount of energy reserves relative to structural size, is a physiological unit of considerable interest in conservation-focused bioenergetic studies. As the physical manifestation of energy balance, body condition provides essential information about the health of individuals and populations (Brock *et al.,* 2013; Williams *et al.,* 2013; Christiansen *et al.*, 2020; Raverty *et al.*, 2020; Stewart *et al.*, 2021a). A variety of metrics have been developed to estimate body condition of free-ranging marine mammals, such as those derived from blubber measures, morphometrics, biochemical or chemical markers, body composition and omics (see reviews in Bowen and Northridge, 2010; Castrillon and Bengtson Nash, 2020). A universal metric does not currently exist for marine mammals due to their differences in life -history traits, habitat use, accessibility, body morphology, and the dynamics of energy storage and utilization (Noren and Wells, 2009; Noren *et al.,* 2015, 2021; Kershaw *et al.,* 2017; Castrillon and Bengtson Nash, 2020; Larrat and Lair, 2022). Even within a species, different metrics may be needed depending on the disposition (e.g. free-ranging vs. stranded) and state (e.g. reproductive status) of the animal. Thus, metrics often need to be validated (e.g. Noren *et al.,* 2019) for individual species or groups when possible. Within the context of bioenergetics, metrics that are comparatively inexpensive and non-invasive (i.e. do not require animal handling or tissue sampling) are likely to be the most useful, such as the recent use of unmanned aerial vehicles to estimate body morphology (see What tools do we have to address these key questions?). Such approaches allow body condition data to be collected from many individuals of all age and size classes, reproductive states and body conditions with minimal disturbance.

### How does body condition relate to survival and reproductive success? (Q8, Q9)

Behavioural changes are one of the first observable responses of individuals to disturbances or environmental perturbations. The resulting impact of that behavioural change on survival and reproductive success is what drives population dynamics (Pirotta *et al.,* 2018a). As illustrated in the PCoD framework (Pirotta *et al.,* 2018a, Fig. 1), exposure to stressors may affect vital rates through bioenergetic (e.g. changes in energy stores) or (mostly) non-bioenergetic pathways (e.g. immune function, contaminant burden). There also may be feedback between these pathways, such as when a disturbance alters energy balance, which then leads to reduced immune function (or vice versa; Brock *et al.,* 2013; Vera-Massieu *et al.,* 2015). While the focus here is on bioenergetic pathways, relationships between body condition and vital rates may thus incorporate the effects of non-bioenergetic pathways as well. In marine mammals, body size and condition metrics are positively related to foetal growth (Christiansen *et al.,* 2014b), pregnancy rates (Williams *et al.,* 2013; Smout *et al.,* 2020), offspring growth (Christiansen *et al.,* 2018) and survival probability (Hall *et al.,* 2001; Beauplet *et al.,* 2005; Harding *et al.,* 2005; McMahon and Burton, 2005; Bowen *et al.,* 2015; Cheney *et al.,* 2018; Oosthuizen *et al.,* 2018; Stewart *et al.,* 2021a). For most marine mammals, these relationships and thresholds of body condition that equate to failed reproduction or imminent mortality remain largely unknown. In addition to the ability to directly link measured body condition from free-ranging animals with vital rates, these relationships (as well as the upper and lower bounds of body condition) are parameters in many PCoD models (Farmer *et al.,* 2018a; Pirotta *et al.,* 2019; Gallagher *et al.,* 2020). Predictions from such models can be sensitive to parameters associated with relationships between body condition and survival (Pirotta *et al.,* 2018b). Even when predicted behavioural patterns are robust to uncertainty in these relationships, the absolute survival values are not (McHuron *et al.,* 2021).

### What is the daily cost of lactation? (Q10)

Lactation is the most costly life -history event that a female mammal will likely experience in her lifetime (Gittleman and Thompson, 1988). Unfavourable environmental conditions (or other changes that affect energy reserves available for lactation) can result in reduced maternal condition or energy delivery to offspring, altered weaning times or longer inter-birth intervals, with different effects depending on reproductive strategy (Trillmich and Limberger, 1985; Arnbom *et al.,* 1997; Noren *et al.*, 2003; Costa, 2008; Chambert *et al.,* 2015; Chinn *et al.,* 2016; Gailey *et al.,* 2020). Knowing how much energy is needed each day to support reproduction is thus key in estimating prey needs and predicting how alterations to energy intake will affect vital rates and population dynamics. The daily cost of lactation has been relatively well studied in pinnipeds using approaches that typically involve repeated handling of individuals, such as combining estimates of milk intake derived from doubly labelled water (DLW) with measurements of milk energy density (e.g. Costa *et al.,* 1986; Iverson *et al.,* 1993; Oftedal *et al.,* 1993; Arnould *et al.,* 1996a; Lydersen *et al.,* 1997; Mellish *et al.,* 1999; Donohue *et al.*, 2002; Wheatley *et al.*, 2006; McDonald *et al.,* 2012). Empirical attempts to estimate lactation costs in most other marine mammals, particularly cetaceans, have been hampered by the inability to quantify milk intake. Relative costs of lactation have been estimated for free-ranging southern right whales (*Eubalaena australis*) by measuring changes in body size and condition of lactating females relative to the growth of their dependent calves (Christiansen *et al.,* 2018), an approach that requires an estimate of the female’s FMR. Other studies have relied on data derived from captive individuals (Williams *et al.,* 2011) or by summing the estimated costs experienced by a dependent calf (Fortune *et al.,* 2013; Villegas-Amtmann *et al.,* 2015).

**Figure 4:**
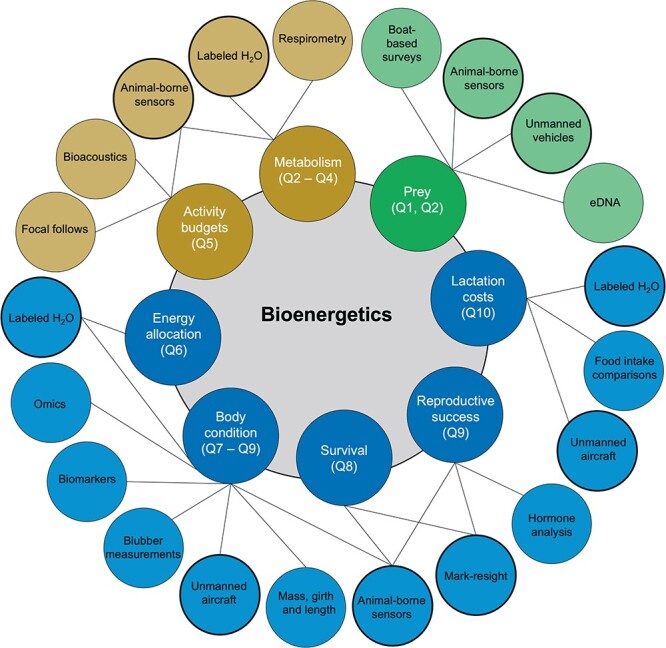
Examples of common and emerging tools or methodologies that can be used to address the key bioenergetic questions identified as part of this exercise. Questions (excluding Q11) are colour-coded as in Fig. 3, with black lines connecting the question to the tool/methodology. Bolded outlines correspond to tools/methodologies that are linked to multiple key questions. In some instances, these represent different tools for collecting the same type of data (e.g. unmanned vehicles and boat-based surveys using acoustic data to characterize prey landscapes), whereas in others they represent different methodologies to address the same question (e.g. eDNA vs. boat-based surveys to characterize prey landscapes).

### How do we best extrapolate data from one species or population to another? (Q11)

Marine mammals are notoriously difficult to study in a natural setting due to logistical challenges that prevent direct measurement. Simple bioenergetic questions, such as ‘how much does an animal weigh?’ or ‘how much prey does an animal consume?’, often require complex or imaginative solutions as most species are either too large to handle or spend most of their time in remote areas underwater. Researchers must extrapolate from the best available biological knowledge when data are missing for their species of interest, with little formal guidance to help inform decisions. Should we look to a closely related species even though they may not share similar body sizes or ecological roles? Should we prioritise habitat use and behavioural similarities over phylogeny? Or should we rely on anecdotal observations from the species of interest even though they may be based on observations from just a few individuals? Choosing the correct input parameters for bioenergetic models has real-world consequences for developing quality management decisions and conservation policy. Several recent meta-analyses have explored variation in metabolic rates (Costa and Maresh, 2017), milk intake (Riek, 2008, 2011, 2021) and lactation strategies (Schulz and Bowen, 2004). Further efforts are needed to understand species groupings across a suite of parameters that are influential on bioenergetic model outputs (Winship *et al.,* 2002; New *et al.,* 2013; Bejarano *et al.,* 2017; Gallagher *et al.,* 2018; McHuron *et al.,* 2020), and whether the appropriate proxy varies depending on the parameter of interest.

### What tools do we have to address these key questions?

Many of the methods or tools needed to address these key questions have been around since the inception of the field of marine mammal bioenergetics (Fig. 4). Labelled water, a method that was pioneered in the 1950s (Lifson and McClintock, 1966), remains one of the most direct measures of FMR, milk intake and body composition, and is still widely used for these purposes (e.g. Mellish *et al.,* 1999; Arnould *et al.,* 2003; Fowler *et al.,* 2007; Lang *et al.,* 2011; Pagano *et al.,* 2018). This approach, however, has limited application to cetaceans given logistical constraints surrounding sample collection and potential violation of the assumption that no seawater is ingested during the measurement period (Hui, 1981). Observations of marked individuals throughout their lifetime, such as those obtained from long-term research programs, have provided a wealth of information on reproductive success and survival (e.g. Ford *et al.,* 2010; Schwarz *et al.,* 2013; Wells *et al.,* 2014; Bowen *et al.,* 2015; Le Boeuf *et al.,* 2019). It is difficult to envision how questions that relate bioenergetics to survival and reproductive success could be answered without continued support for such efforts.

Emerging technologies and innovative solutions have played a pivotal role in our ability to answer some of these key questions (Fig. 4). For example, animal-borne sensors, and associated statistical approaches for analysing data, have become an invaluable tool for addressing a wide range of questions identified as part of this exercise (Fig. 4). New sensors facilitate data collection on prey capture (e.g. Tennessen *et al.,* 2019; Olivier *et al.,* 2022), drift rates (used to infer body condition; e.g. Biuw, 2003; Biuw *et al.,* 2007) and acceleration, breath rate and heart rate (used to infer FMRs; e.g. Isojunno *et al.,* 2018; Wilson *et al.,* 2020; McDonald *et al.,* 2021). They also provide information on the physical and biological environment that can characterise the prey landscape (Arranz *et al.,* 2011; Goulet *et al.,* 2019; McMahon *et al.,* 2019, 2021). Unmanned systems are currently being applied to marine mammal bioenergetics, particularly the use of aerial systems to estimate body condition and growth (Fearnbach *et al.,* 2018; Lemos *et al.,* 2020; Aoki *et al.,* 2021; Currie *et al.,* 2021; Shero *et al.,* 2021; Stewart *et al.,* 2021a; Christiansen *et al.,* 2022), and surface and underwater systems to survey prey communities (Kuhn *et al.,* 2019; Benoit-Bird *et al.,* 2020). Environmental DNA (eDNA) is a promising emerging tool for addressing questions related to prey landscapes, as it appears able to characterise the distribution and diversity of prey communities (Visser *et al.,* 2021) as well as prey biomass (Rourke *et al.,* 2022). While still in its infancy, eDNA has been used to quantify prey distribution and diversity in areas with critically endangered populations of Yangtze finless porpoise (*Neophocaena asiaeorientalis*; Qu *et al.,* 2020) and to detect spatiotemporal variability in pelagic forage fish in the Saguenay–St. Lawrence Marine Park, an area used by endangered beluga whales (*Delphinapterus leucas*; Berger *et al.,* 2020).

Regardless, some key questions are unlikely to be comprehensively addressed without further technological advancements or validation. In particular, the question about FMR, which was the most agreed-upon key question by survey participants, is one area where both validation and advancements are needed. For example, there remains uncertainty in how the approaches commonly used to estimate, or infer, cetacean FMRs (e.g. breathing rates) compare with methods that provide a more direct measure of FMR (e.g. DLW) and the level of uncertainty around estimates given the assumptions of such approaches (Fahlman *et al.,* 2016 and associated responses). New methods that provide estimates or broad scale proxies of FMR from tissue samples (e.g. Chung *et al.,* 2019) would be extremely valuable in furthering our understanding of FMRs in marine mammals, particularly baleen whales and deep-diving odontocetes. Non-invasive sensing technology using near-infrared spectroscopy that continuously measures the rate of O_2_ consumption is being developed, which provides a new avenue to understand energetic regulation in marine mammals (Ruesch *et al.,* 2022). Other areas where advancements are needed include continuing efforts to improve animal-borne sensors (e.g. miniaturisation, data transmission and processing) and validation of existing and development of new approaches for estimating body condition and lactation costs of cetaceans.

## Concluding remarks

Marine mammals tend to live in spatially and temporally variable environments that are changing rapidly with recent climate changes. At the same time, they are also facing increasing exposure to human activities in the marine environment. Such conditions will continue to influence individuals and populations through bioenergetic pathways, which can have cascading impacts on the ecosystems they inhabit through consumptive or non-consumptive mechanisms (Kiszka *et al.,* 2015; Estes *et al.,* 2016; Savoca *et al.,* 2021). Changing environmental conditions may not always negatively impact species, and in some cases may facilitate range expansion, which could alter consumptive pressure on prey populations and create new management challenges. Here we have identified 11 key questions that may help guide research priorities to further our understanding of these pathways. While comprehensive, this list of key questions is certainly not exhaustive and does not necessarily imply that questions that were not included are unimportant or should not be addressed. Instead, they represent the questions that most participants agreed were important gaps, indicating that addressing these questions might have the broadest application across different disciplines, species and approaches.

The end goal of many marine mammal bioenergetic studies is to provide information that can be used to inform policies to better conserve populations by minimising or mitigating risks from human activities in the context of ecosystem management. For example, research on energy requirements, prey intake and body condition of Southern Resident killer whales has contributed to management decisions aiming to ensure adequate Chinook salmon (*Oncorhynchus tshawytscha*) availability to aid in killer whale population recovery (Ford *et al.,* 2010; Noren, 2011; Williams *et al.,* 2011; Chasco *et al.,* 2017; Wasser *et al.,* 2017; Stewart *et al.,* 2021a). Similarly, PCoD modelling of sperm whales (Farmer *et al.,* 2018a) was used to inform NOAA’s Biological Opinion on federal oil and gas program activities in the Gulf of Mexico. In addition to providing information about the health of marine mammal populations and insights into factors that may be affecting population trajectories, the data collected to address the key questions identified in this exercise will help refine and verify the values of the parameters used in bioenergetic models. This will ensure more accurate predictions of energy needs and the consequences of anthropogenetic and environmental impacts on marine mammal populations.

## Funding

This work was funded by the Marine Mammal Commission (MMC19-173). The Office of Naval Research funded the bioenergetic workshop (N000142012392) that provided support for this work.

## Data availability

The original submitted questions and final collated questions are included in supplemental material. There are no other data associated with this article.

## Supplementary Material

suppl_coac055
